# Prevalence of hypertension in community members in a low-income community in Windhoek, Namibia

**DOI:** 10.4102/phcfm.v17i1.4735

**Published:** 2025-04-18

**Authors:** Zelda Janse van Rensburg, Craig Vincent-Lambert

**Affiliations:** 1 Department of Nursing, Faculty of Health Sciences, University of Johannesburg, Johannesburg, South Africa; 2 Department of Emergency Medical Care, Faculty of Health Sciences, University of Johannesburg, Johannesburg, South Africa

**Keywords:** non-communicable diseases, prevalence, hypertension, Namibia, low-income, peri-urban

## Abstract

**Background:**

Hypertension remains a significant risk factor for the development of several non-communicable diseases such as stroke, myocardial infarction and renal failure. In many African countries, undiagnosed and unmanaged hypertension within the population remains a challenge. Proactive screening and health education therefore become important interventions.

**Aim:**

This study aimed to investigate the prevalence of hypertension among community members in a low-income, peri-urban community.

**Setting:**

The study was conducted in Otjomuise township, Windhoek, Namibia.

**Methods:**

A quantitative, cross-sectional design with a survey method was followed. A pre-validated health screening instrument was used to record the presence of hypertension in a sample of 358 community members who presented for screening over 2 days. A purposive sampling method was employed. Systolic and diastolic blood pressure reading were recorded, analysed and categorised using the American Heart Association classification scale. Blood pressures were classified as being either normal, elevated, hypertension stage 1 or hypertension stage 2. The data were analysed by a statistician using SPSS version 27 statistical software program and presented in tables.

**Results:**

A total of 210/358 (59%) of participants were found to have blood pressures within normal limits. Moreover, 57/358 (16%) had elevated blood pressures, 52/358 (14%) were in hypertension stage 1 and 39/358 (11%) were in hypertension stage 2.

**Conclusion:**

The 41% prevalence of abnormally elevated blood pressures found in our study is in line with the findings of other studies and confirms that hypertension in the Namibian population remains an ongoing public health concern. Larger scale and more regular screening exercises with appropriate referral are recommended.

**Contribution:**

Our study give insight on the prevalence of hypertension in a community in Namibia.

## Introduction

Hypertension is often asymptomatic and referred to as the ‘silent killer’.^1^ Hypertension is so significant that certain authors have reported this as the greatest single contributor to the global burden of disease and mortality with about 10.8 million deaths occurring in 2019 being attributed to this disease.^1,2^ Hypertension is a risk factor for cardiovascular and circulatory diseases, including stroke, myocardial infarction and renal failure.^3^ Coronary heart disease and stroke cause approximately 9.4m deaths each year globally.^4^

Most of the estimated 1.13 billion people suffering from hypertension reside in low-middle income countries, with the highest prevalence rates seen in Africa.^5^ The prevalence of hypertension in Africa is estimated at 46%^6^ among those 25 years and older, between 55.2% and 57.0% among older adults aged 50 to 55 years and 66.7% among those aged 60 years and older.^5^ In sub-Saharan Africa (SSA), the prevalence of hypertension has been reported to be as high as 48% in women and 34% in men in 2019.^2^

Namibia is a middle-income country in SSA with an estimated population of 2.61m people in 2023.^7^ In 2022, 59.7% of the Namibian population were between the ages of 15 and 64 years,^7^ followed by 36.2% between the ages of 0 and 14 years. Only 3.9% of the total Namibian population were over the age of 65 years, making Namibia a relatively ‘young’ country. Non-communicable diseases (NCDs) including cardiovascular disease, diabetes, and cancer contribute to nearly 41% of all annual deaths in Namibia with hypertension being identified as a significant contributor.^4^ In 2019, the World Health Organization (WHO) reported that 44% – 46% of the total Namibian population was suffering from hypertension.^2,5,8^ Despite the high prevalence of hypertension in Namibia, the disease remains largely undiagnosed and untreated.^8^ Even when diagnosed, the effectiveness of treatment interventions for hypertensive cases remains poor with only 18% of males and 25% of females diagnosed with hypertension being considered well controlled.^8^

There are several risk factors that contribute to the development of hypertension including age, tobacco use, obesity, inactivity and alcohol consumption.^8^ Other risk factors are diet-related including a high intake of salt, sugar, and fat and a low intake of fruits and vegetables.^2^ In SSA, the transition from a traditional lifestyle such as eating organic and self-planted fruits and vegetables to a more westernised lifestyle such as eating processed food is also a major contributing factor for hypertension.^2^

Research conducted in the capital city of Windhoek through several household surveys showed that, along with high levels of food insecurity, many community members lack diversity in their diet and are largely dependent on starchy staples, sugars and foods made from oils with an inadequate consumption of healthier foodstuffs such as fruits and vegetables.^9^ This type of diet can contribute to people becoming overweight and obese with an increased risk of developing hypertension or other NCDs.^9^

The WHO global target for the prevention and control of NCDs globally (2013–2020) was aimed at a 25% relative reduction in the overall mortality from cardiovascular diseases, cancer, diabetes, or chronic respiratory diseases by 2025.^10^ The Sustainable Development Goals (SDG) target 3.4 is set at a one-third reduction in premature deaths from NCDs by 2030.^10,11^ Such targets exist in a context where NCDs are viewed as both preventable and manageable, provided the causative disorders and drivers can be detected and managed early on. This is important in the context of our study as hypertension remains both preventable and treatable with early detection and the patient’s adherence to prescribed antihypertensive treatment interventions.^1^

Despite the above being well documented and known, the early diagnosis, treatment and adherence to treatment interventions remain a significant challenge among many African countries and hamper their achievement of the WHO goals.^5,10^ Also, the level of development of the country, region and the socioeconomic context can play a significant role in the effectiveness of efforts to manage hypertension. In Namibia, there are free hypertension screening and treatment programmes administered under the Ministry of Health and Social Services (MoHSS).^5^

In this article we aim to share our findings about the prevalence of hypertension in a sample of community members in a low-income, peri-urban community in Windhoek, Namibia and make selected recommendations for future research and related interventions that may assist to better understand the nature of hypertension in African contexts and assist in management of the disease.

## Research methods and design

A quantitative, cross-sectional design with a survey method was followed using a pre-validated health screening instrument. The community outreach project was funded by the University of Johannesburg.

### Study setting

The community of Otjomuise is a low-income, peri-urban community in north-west Windhoek, Namibia. Adjacent to the residential area of Otjomuise, is a large informal settlement named ‘Agste laan/Eight avenue’. The community is characterised by poverty, high unemployment rates, substance abuse and scarce resources such as running water and electricity. In 2020, Windhoek had a total of 41 900 informal housing dwellings or ‘shacks’, accommodating close to 100 000 residents.^12^ Data describing the exact population of Otjomuise could not be found, however the total population of Windhoek at the time of the study was around 3 022 401 people.^7^ Otjomuise government Primary Health Care (PHC) clinic is located within the community of Otjamuise, with the nearest government hospital, Katatura Hospital in Khomas being 10 km away.^7^ The data collection took place just outside Otjomuise PHC clinic, within the clinic grounds. The setting was convenient as many community members passed the clinic on the way to work or home and could easily be approached to participate in the free screening. Those who attended the clinic were also encouraged to present themselves for the free screening after their consultation.

### Population, sample and sampling strategy

The target population consisted of adult community members of the Otjomuise community, aged 18 years and older. The Otjomuise community was purposively selected by academics from the Namibia University of Science and Technology who were familiar with the socio-economic conditions and health-related challenges in this community. The sample consisted of 358 community members who attended the screening and who provided informed consent for their demographic and health data to be captured and included in the study.

### Data collection and instrumentation

The screening was conducted in July 2023. Although health screening such as blood pressure, cholesterol and glucose screening are offered to most patients at Otjamuise clinic, visual screening are not routinely offered to all patients. The nurses referred patients for screening and the community health workers used word of mouth and direct engagement strategies to invite members of the community to present themselves for free health screening over the 2 days that data was gathered. Those who did not meet the inclusion criteria (being an adult aged 18 and above) and those who did not agree to participate were still offered an opportunity to receive the free screening; however, their data were not included in the study. Where there were significant concerns with either the systolic blood pressure (SBP), diastolic blood Pressure (DBP) or both, the respondent was counselled and referred to the nearest clinic (Otjamuise clinic or Katatura Hospital) for further investigation and treatment.

A survey adapted from the WHO^13^ STEPS questionnaire was used to capture the results of the screening^13^ We added a section to capture selected demographic data including gender, age, highest level of education, marital status, and employment status.

Participants had their blood pressures measured using a Microlife^®^ electronic non-invasive blood pressure (NIBP) monitor. Both SBP and DBP were taken at three different times. All blood pressure recordings were measured on the right arm after a 5-min rest period. The NIBP monitors were calibrated before use and checked again before taking the blood pressure levels. Participants who presented with abnormal readings were allowed to ‘rest’ for about 30 min and have their blood pressure taken again for a fourth time at the end of the screening process to verify the accuracy of readings.

### Data analysis

Data from the screening forms were uploaded onto an Excel spread sheet. Separate sheets were used to record the socio-demographic data, the history taking and the blood pressure readings. Other results such as the respondent’s height, weight and body mass index (BMI), cholesterol and blood glucose levels and eye screening were also recorded; however, these data are not the subject of discussion in this article. The data were analysed by a statistician using SPSS version 27 statistical software program. The data were presented as descriptive statistics using tables. The blood pressure classification and ranges from the American Heart Association were used to report on the prevalence of hypertension in this sample of community members and are indicated in Table 1.^14,15^

**TABLE 1:** American Heart Association classification of hypertension based on blood pressure measurements.

Blood pressure classification	SBP	DBP
Normal BP	< 130 and	< 85
Elevated	130–139 and/or	85–98
High blood pressure (hypertension stage 1)	140–159 and/or	90–99
High blood pressure (hypertension stage 2)	≥ 160 and/or	≥ 100

*Source*: Adapted from Unger T, Borghi C, Charchar F, et al. 2020 International Society of Hypertension Global Hypertension practice guidelines. Hypertension. 2020;75(6):1334–1357. https://doi.org/10.1161/HYPERTENSIONAHA.120.15026; Van Rensburg ZJ, Vincent-Lambert C, Razlog R, Phaladze N. Prevalence of hypertension in a sample of community members in a low-income peri-urban setting in Gaborone, Botswana. J Public Health Afr. 2023;14(2):2068. https://doi.org/10.4081/jphia.2023.2068

SBP, systolic blood pressure; DBP, diastolic blood pressure; BP, blood pressure.

### Ethical considerations

Ethical clearance to conduct this study was obtained from the University of Johannesburg Research Ethics Committee (No. REC-1985-2023). Permission was also granted from the Namibia Ministry of Health and Social Service to conduct the study. Written consent was obtained from each respondent before the screening was conducted and a respondent number was allocated to each respondent to maintain confidentiality of the respondent and to protect their privacy. The respondents were free to decide whether they wanted to participate or not and were not forced in any way. If a respondent started the screening but opted to terminate the screening, the respondent was free to do so without any negative consequences.

## Results

Our results are presented beginning with the participants’ demographics (Table 2). Thereafter, in Table 3, we highlight the prevalence of hypertension within the sample.

**TABLE 2:** Demographic data of the study participants.

Variable	Frequencies (*n*)	Percentage (%)
**Age (years)**		
18	18	5.0
19–29	82	22.9
30–39	69	19.3
40–49	86	24.0
50–59	69	19.3
60–69	28	7.8
70–79	3	0.8
80–89	3	0.8
Total	358	100.0
**Gender**		
Male	116	32.4
Female	242	67.6
Total	358	100.0
**Highest level of education**		
No formal schooling	26	7.3
Less than primary school	18	5.0
Primary school completed	95	26.5
Secondary school completed	122	34.1
High school completed	64	17.9
College and/or university completed	23	6.4
Postgraduate degree	2	0.6
Refused to answer	8	2.2
Total	358	100.0
**Marital status**		
Never married	241	67.3
Currently married	78	21.8
Separated	12	3.4
Divorced	12	3.4
Widowed	10	2.8
Cohabitating	2	0.6
Refused	3	0.9
Total	358	100.0
**Employment status**		
Government employee	24	6.7
Non-government employee	63	17.6
Self-employed	63	17.6
Student	51	14.2
Homemaker	17	4.7
Retired	12	3.4
Unemployed (able to work)	99	27.7
Unemployed (unable to work)	28	7.8
Refused	1	0.3
Total	358	100.0

### Demographics

The demographic data showing the participants’ gender, age, highest level of education, marital and employment status is presented in Table 2.

As shown in Table 2, most of the respondents were between the ages of 19 and 59 years (66.2%). Few respondents were 60 years and above (9.4%) or 18 years of age (5%). Our participants were mostly females (67.6%). Most of our participants, 78.5%, indicated that they completed either primary school (26.5%), secondary school (34.1%) or high school (17.9%). Few respondents indicated that they had no formal schooling (7.3%), or schooling less than primary school completed (7.8%). Most of our participants were not married (67.3%). A total of 27.7% of our participants indicated that they were unemployed while they were able to work and another 7.8% indicated that they were unemployed but unable to work. Most of our participants were non-government employees (17.6%), self-employed (17.6%) or a student (14.2%).

### Prevalence of hypertension

The blood pressure of the study participants (*N* = 358) is indicated in Table 3.^14,15^

**TABLE 3:** Blood pressure measurements according to categories.

Blood pressure classification	SBP	DBP	Frequency (*n*)	Percentage (%)
Normal BP	< 130 and	< 85	210	59
Elevated	130–139 and/or	85–98	57	16
High blood pressure (hypertension stage 1)	140–159 and/or	90–99	52	14
High blood pressure (hypertension stage 2)	≥ 160 and/or	≥ 100	39	11

**Total**	**-**	**-**	**358**	**100.0**

*Source*: Adapted from Unger T, Borghi C, Charchar F, et al. 2020 International Society of Hypertension Global Hypertension practice guidelines. Hypertension. 2020;75(6):1334–1357. https://doi.org/10.1161/HYPERTENSIONAHA.120.15026; Van Rensburg ZJ, Vincent-Lambert C, Razlog R, Phaladze N. Prevalence of hypertension in a sample of community members in a low-income peri-urban setting in Gaborone, Botswana. J Public Health Afr. 2023;14(2):2068. https://doi.org/10.4081/jphia.2023.2068

SBP, systolic blood pressure; DBP, diastolic blood pressure; BP, blood pressure.

From Table 3, one can see that 210/358 (59%) of our participants had a normal BP while 57/358 (16%) had elevated blood pressures with 52/358 (14%) being classified as hypertension stage 1 and 39/358 (11%) as hypertension stage 2.

## Discussion

### Demographic data

As one ages, the risk of developing hypertension increases.^16^ Age is linked to factors such as hardening and shrinking of arteries.^17^ A 2015 study conducted Namibia’s neighbouring country Botswana found that people between the ages of 55–64 years were seven times more likely to have hypertension compared to those aged 25–34 years.^16^ Interestingly, only 30% of our participants were older than 50 years. Reasons for this are not known, however a similar study conducted in Botswana also revealed that few community members over the age of 50 years presented themselves for free blood pressure screening.^18^ It is possible that the prevalence of hypertension found within our sample may have been even higher if more community members over the age of 50 years had participated.

There were significantly more females than males who presented themselves for the free screening. This was expected as globally females are more prone than males to seek healthcare and health screening.^18^ At the time of the study, the female population was slightly higher at 1 548 177 females as compared to 1 474 224 males. This indicates that there were 73 953 more females in the city of Windhoek. Although there was not a significantly large difference between the number of females and males, this may have been a reason for the underrepresentation of males in the study. Because men usually work during the day as the breadwinner of the family, the uptake of free health screening by men is influenced by factors such as health beliefs, fear of knowing that they have a disease and the fear of being viewed as ‘weak’ or not masculine.^19^

Only 64 (17.9%) of our participants indicated that they had completed high school and very few (6.4%) indicated having a college or university education (0.6%). Previous studies have linked lower levels of education to the propensity to develop hypertension.^20^ This linkage has been attributed to low literacy and low health literacy levels.^21^ Similarly, a study conducted in 2014 in South Africa found that women with higher levels of education had lower levels of blood pressure.^21^

Factors such as stress caused by work or unemployment may be a risk factor for development of hypertension.^2,10,22^ Stress related to elevated blood pressure is, however, thought to be more prevalent among men than women.^2^ In our study, 35.5% of our participants indicated they were unemployed with 17.6% indicating that they were self-employed. A similar study conducted in Botswana with similar results regarding elevated blood pressure, revealed a 39% unemployment rate among its participants.^15^

Regarding marital status, 67.3% of our participants indicated that they were unmarried. Evidence shows that having a partner with hypertension increases one’s risk of having high blood pressure.^3^ Another study however indicated that the prevalence of hypertension increases among those who are married.^23^

### Prevalence of hypertension

Hypertension is a growing concern in Africa and Namibia appears to be no exception with a previous study revealing a 46% prevalence of hypertension.^2^ Our study had a similar finding with a 41% prevalence of elevated blood pressure levels being found within our sample of community members tested. This is slightly more than the 36% found in a similar study conducted in a similar context in Botswana.^16^ Other studies conducted in Africa found the prevalence of hypertension to range from 13% to as high as 42%.^15^

Regarding the classification of hypertension we found, 16% of our participants presented with an elevated blood pressure, 14% with hypertension stage 1 and 11% with hypertension stage 2. These findings are in line with the results published by the WHO in 2019^24^ that reported a 44% – 46% prevalence of hypertension in Namibia.^24^ The results are also in line with other studies that reported on the prevalence of hypertension in countries such as India (40%), the Unites States of America (44%)^4^ and Taiwan (35%).^25^

The fact that most of the hypertensive cases we found fall into the elevated (16%) or hypertension stage 1 category is potentially good news as early detection of elevated or stage 1 hypertension together with appropriate intervention can significantly decrease the progression to hypertension stage 2 and related systematic complications.^8^ This becomes an important finding as it is estimated that there is a 30% conversion rate of hypertension stage 1 to hypertension stage 2 every 4 years,^22^ and hypertension stage 2 has a high risk of organ damage.^26^ Cardiovascular complications such as myocardial infarction, heart failure, stroke and renal failure are also related to hypertension stage 2.^27^

### Study’s limitations

The nature of our recruitment made it difficult to control the sample size and the percentage of males and females that presented themselves for the screening. As most of our participants were between the age of 18 and 50 years, the prevalence of stage 1 and specifically stage 2 hypertension may have been greater if older community members had presented for the screening. Comorbidities such as diagnosed hypertension and diabetes were not taken into consideration when the screening was performed. Comorbidities might have influenced the prevalence of hypertension among the community members. Although the respondents were given time to rest before the screening, factors that may cause increased blood pressure, such as stress and anxiety, were not ruled out beforehand. The blood pressure equipment was calibrated beforehand, and all students received training on the measuring and documentation prior to the screening. Information bias might however have occurred and influenced the accuracy of the data that were collected; however, none of the results suggested any biasness.

## Conclusion

In our study, we did not focus heavily on the recording of detailed information about behavioural risk factors such as alcohol consumption, physical inactivity and diet. Future studies of this nature may consider also recording these predictors of hypertension.

Based on what we found, we would recommend healthcare managers consider more regular screening exercises that revisit the same community over time to continue to identify and refer hypertensive cases and importantly to establish what trends are developing. This should be coupled with the provision of additional health education and advice to community members about the nature of hypertension and how this may be managed through diet, exercise and regular health checks.

The SDG target 3.4 is aimed to reduce premature mortality from NCDs by a third by 2030 relative to 2015 levels (SDG countdown). Even though this target is in place, NCDs continue to be the leading cause of morbidity and mortality globally (SDG countdown). The prevalence of hypertension among Namibian adults remains high, despite free healthcare in the country. Future research should be targeted to determine interventions needed to improve the overall diagnosis and prevention of hypertension in Namibia.
